# Understanding pathways to recovery from alcohol use disorder in a Black community

**DOI:** 10.3389/fpubh.2025.1537059

**Published:** 2025-05-01

**Authors:** Adam Vose-O’Neal, Shanesha Christmas, Karen A. Alfaro, Robert Dunigan, Alex P. Leon, Drew Hickman, Andre Johnson, Meelee L. Kim, Sharon Reif

**Affiliations:** ^1^Institute for Behavioral Health, Heller School for Social Policy and Management, Brandeis University, Waltham, MA, United States; ^2^Detroit Recovery Project, Detroit, MI, United States

**Keywords:** recovery science, recovery pathways, recovery stages, Black/African American, racial equity, alcohol use disorder, addiction

## Abstract

**Introduction:**

Black Americans suffer a range of health disparities rising from a long history of structural inequities and racism. Black individuals experience alcohol use disorder (AUD) at rates comparable to the general population, yet they suffer more negative consequences due to alcohol use such as illnesses, injuries, criminal-legal involvement, and social problems. The barriers they face challenge their ability to achieve recovery. However, the recovery needs of the Black population and the potential impact of racial disparities on pathways to recovery have not been examined.

**Methods:**

We conducted semi-structured interviews with 37 participants in the Black-majority city of Detroit, Michigan, who identified as Black or African American and in recovery from AUD. Participants were 50 years old on average, 40% were women, and they reported being in recovery from alcohol for 8.4 years on average. We built upon *a priori* codes, using a framework analysis approach, to identify and code thematic domains related to recovery pathways.

**Results:**

We identified four overarching themes. (1) *Delayed recovery initiation* largely due to systemic challenges and a lack of knowledge about recovery, resulting in the belief that recovery was not possible. (2) Once initiating recovery, many reported getting stuck in *chronic early recovery* due to relapse cycles that regularly involved system and individual challenges coupled with inadequate support. (3) Use of blended *recovery pathways*, some common in the recovery literature (e.g., Alcoholics Anonymous), and some more prevalent in Black communities (e.g., religion/spirituality). (4) The *facilitators of recovery* var*y by recovery stage*; for example, receiving support was crucial in early recovery while providing support was important for sustained recovery.

**Discussion:**

Participants’ stories emphasized the burdens experienced by this low-income Black community at personal, interpersonal, environmental and societal levels. They directly connected these burdens with the difficult mission of achieving and sustaining recovery from alcohol problems. Some challenges and recovery pathways were common in the broader population, and some, such as the impact of racism, were unique to this Black population. The results have meaningful implications for clinical treatment and recovery support improvements, to advance the recovery journeys of Black individuals with AUD.

## 1 Introduction

Despite being less likely to drink alcohol, starting to drink later, and misusing alcohol less frequently than other racial and ethnic groups, Black Americans experience alcohol use disorder (AUD) at rates comparable to the general US population (1–4). Further, Black individuals suffer more negative consequences due to alcohol use such as illnesses, injuries, criminal-legal involvement, and social problems (5–8). Black individuals also encounter more barriers to accessing mental health and substance use treatment than white individuals (9, 10) and are less likely to initiate alcohol or drug treatment (11).

Black Americans demonstrably suffer from a range of health disparities rising from a long history of structural inequities and racism (12–15). For example, if Black individuals do initiate and complete substance use treatment, they have worse health outcomes than their white counterparts (16–18). These disparities have been attributed, in part, to differences in socioeconomic status, housing stability, and transportation access (19, 20). Implicit racial bias among health care providers (21), differential treatment of minorities (16, 22) and mistrust of the medical establishment (23, 24) also play a role. Treatment is a major pathway to recovery for over half of people who have resolved an alcohol or drug problem (25), thus these structural barriers likely impede recovery for many Black individuals.

Recovery is a dynamic process of behavior change leading to stable improvements in functioning, sense of purpose and well-being (26–30). With a few exceptions (27, 28, 31, 32), little is known about the combination of factors that contribute to recovery, in part because the recovery process is unique to individuals. One way of examining recovery pathways is to consider the stages of recovery; that is, what happens as someone progresses from initial to long-term recovery. Recovery stage models have usually included three or four stages—e.g., initial, early, sustained, and stable (33–36)—and have attempted to map key recovery themes and characteristics by stage (33, 35). For example, we know that those in the early stages of recovery are particularly vulnerable to relapse (37), thus recovery support services and social supports are important at this stage. How recovery occurs and is sustained may also vary by the substance of choice (25). For instance, alcohol is a legal substance, widely available, and commonly used in many social settings, and thus may be harder to avoid while working to attain and sustain recovery (37).

Racism and structural discrimination have led to challenges for many Black individuals beyond the health and healthcare impacts already mentioned and, in turn, are associated with alcohol and drug use and addiction and interact with already prevalent barriers to seeking care (9, 24, 38, 39). For example, Black people in the US experience poverty at more than twice the rate of white individuals (40). Redlining in urban areas such as Detroit resulted in entrenched neighborhood segregation that remains today (41). Alcohol outlets are much more prevalent in low-income Black neighborhoods, as is violence and crime associated with alcohol and drugs (8, 42–44). Experiences of limited employment opportunities, inadequate housing, poor mental health, trauma, intergenerational trauma, and resulting hopelessness, particularly in low-income Black communities, are frequent drivers of substance use (45–48). Treatment and recovery services that are supportive of the Black experience in the U.S. should be able to counter some of these effects and build recovery capital (49–51). In particular, spirituality and religion is an important component associated with healing and well-being in the Black community and may serve as a coping resource to improve the lives of Black individuals with alcohol problems (50, 52–54).

Nationally representative data suggest that, among people who reported alcohol or drug problems, people who identify as Black had similar rates of being in recovery as people who were white or Hispanic (1). However, the barriers faced by many Black individuals likely challenge their ability to achieve and sustain recovery from substance use, either assisted or unassisted. The recovery needs of many in Black communities and the potential impact of racial disparities on pathways to recovery have not yet been examined. Moreover, no research has focused on the heterogeneity within Black populations, which is why this study takes a “centering the margins” approach (55). To understand recovery pathways in the Black community, particularly in a low-income urban area, we must start with understanding the people and who they are before, during, and after experiences with alcohol problems, including a consideration of the broader racialized context. We used primary data collection to begin this process of understanding pathways among Black individuals in recovery from AUD. By allowing individuals to tell their stories of addiction and recovery in their own way, themes arose that were unique to this Black community and their experiences, providing a nuanced understanding of recovery.

## 2 Methods

### 2.1 Study overview

The qualitative findings reported in this paper are part of the UPWARD project (Understanding Pathways to Wellness and Alcohol Recovery in Detroit), a mixed methods study of pathways to recovery from AUD in a Black community. Here, we explore the experiences and perspectives of Black participants’ AUD recovery pathways, emphasizing the heterogeneity of the Black population in AUD recovery. The study was approved by Brandeis University’s Institutional Review Board (IRB). We followed the Consolidated Criteria for Reporting Qualitative Research (COREQ) for describing the study and results (56).

### 2.2 Approach

A semi-structured interview format allowed for conversational flexibility, which enabled us to understand each participant’s unique background and recovery story, while maintaining methodological consistency between interviews. The interview guide was adapted from Flaherty et al. (33) who offered a framework for elucidating in-depth recovery stories. Their study explored the addiction recovery pathways of a small number of mostly white participants in long-term SUD recovery. We modified their guide to allow for shorter interviews with Black participants and a wider range of AUD recovery experiences (see Supplementary material). We refined our modified guide through an iterative process that included a literature review, pilot-testing, and research team discussions.

This qualitative component of the study was designed and conducted by a team with over 30 years of collective experience in qualitative behavioral health research. Two team members (A.V.O. and R.D.) hold master’s degrees in social work and collectively have over 15 years of clinical experience; they also are trained as doctoral-level health service researchers. The interview team was diverse regarding race and gender, an intentional attempt to put research participants at ease due to the potential sensitive nature of the subject matter discussed. Participants were asked their preference as to the race and/or gender of their interviewer. R.D., S.C., and A.V.O. conducted the interviews. A.L. and K.A. served as note-takers to supplement the interview recordings.

### 2.3 Setting

The study is set in Detroit, Michigan. Detroit’s population is mostly from minoritized groups, with over three quarters identifying as Black or African American (57). Many Detroit residents are low-income, with 32% living in poverty (57). Racial disparities are especially present in housing, educational attainment, and family income (58). Many individuals have limited or no access to a comprehensive, integrated array of behavioral and physical health services due to a lack of or inadequate health care coverage (58). Detroit generally reflects findings by the CDC, “...residents in mostly minority communities continue to have lower socioeconomic status, greater barriers to health-care access, and greater risk for, and burden of, disease compared with the general population” (58). We collaborated with the Detroit Recovery Project (DRP), a peer-led and peer-driven community organization whose mission is to support recovery which strengthens, rebuilds, and empowers individuals, families, and communities who are experiencing alcohol and drug use disorders. DRP offers substance use prevention, treatment and recovery services, with a staff that includes clinical providers and recovery coaches. This collaboration facilitated access to a community of Black individuals who are in recovery from alcohol use disorders.

### 2.4 Recruitment and data collection

Interview participants were recruited from DRP. The study’s on-site research manager (D.H.) informed DRP clinicians and recovery coaches of the project. They, in turn, encouraged their clients to participate. A few participants were recruited directly through posters and flyers at DRP and through snowball sampling. All potential participants were screened by the research manager. To be eligible, participants needed to speak English, be at least 18 years old, identify as being in recovery from AUD (self-reported), and identify as Black or African American. Neither an additional substance use disorder nor active drug use was exclusionary. We tried to capture a range of participant characteristics including gender, age, LGBTQ+ identity, military service, and length of recovery. All participants provided informed consent following a description of the study and an opportunity to ask questions.

Interviews were 40–75 minutes long, conducted through videoconference software. Participants without technical capacity for videoconference were provided private space and the use of a computer at DRP. All interviews were recorded with participant permission, transcribed verbatim, and de-identified by a member of the research team. Participants were compensated ($50) for their time. Interviews were conducted from July 2023 to May 2024.

### 2.5 Participants

Participants were 37 Black or African American adults in recovery from AUD; one participant identified as being multiracial. Fifteen (41%) were female, three (8%) identified as gay, lesbian, or bisexual, and three (8%) indicated active or veteran military status. Most participants (76%) reported being clients at DRP; the remainder reported a connection to DRP (e.g., current or former employee or friends with a client). Participants were an average of 50.2 years old (*SD* = 11.4, range 30–66) at the time of the interview and had been in recovery from AUD for an average of 8.4 years (*SD* = 9.8, range 1 month to 36 years). Most participants (81%) also reported being in recovery from drug use at the time of the interview, for an average of 7.1 years (*SD* = 9.2, range 1 day to 36 years).

### 2.6 Analysis

We identified *a priori* themes, informed by the literature (33, 59, 60). Using a framework analysis approach (61, 62), A.V.O. and S.C. independently coded a sub-sample of transcripts and collaboratively developed a core codebook, grouped by thematic domain, which was then reviewed with A.L., K.A., and R.D. Domains and/or codes were added, subtracted, or amended with agreement from all members of the coding team. We then wrote definitions and precise instructions for each domain and code to increase coding accuracy and consistency. Two team members independently coded each transcript. The entire coding team met regularly to refine the coding process, resolve inconsistencies, re-code as needed, and organize final codes into domains. The results presented here focus on the thematic domains related to recovery pathways. Italics indicate direct quotes, maintaining the language used by participants. The team used ATLAS.ti 24.1.1 to store and analyze the transcripts.

## 3 Results

Our findings are grouped into four overarching themes. First, a common refrain in participant stories was *delayed recovery initiation* due in large part to systemic challenges and a lack of knowledge about recovery, resulting in the belief that recovery was not possible. Second, once initiating recovery, many participants reported getting stuck in *chronic early recovery* due to relapse cycles that regularly involved system and individual challenges coupled with inadequate support. Third, participants described using blended *recovery pathways*, some common in the AUD recovery literature (e.g., Alcoholics Anonymous), and some perhaps more prevalent in Black communities (e.g., religion/spirituality). And fourth, participants described that the *facilitators of recovery* var*y by recovery stage*; for example, receiving support was crucial in early recovery while providing support was important for sustained recovery. Tables 1–4 summarize these themes along with sub-themes and exemplar quotes.

**Table 1:** Delayed recovery initiation.

Sub-theme	Exemplar
Systemic challenges	*I knew a lot of people that their whole family sold dope. They really did not have a chance to do nothing else but sell dope.* *Basically, I was born with the cards dealt against me already.* *Depression, anxiety, trauma goes a lot in the Black community.*
Omnipresence of alcohol and drugs	*You got a liquor store on every corner.*
Individual challenges	*I had a really rough childhood, so I just know I do not want that for my child.* *I think women are scared to come out and seek help.*
Lack of role models	*Learning how to be a young woman was something that took a long time because I just did not have those role models.* *For a long time, I never knew recovery existed.*
Hopelessness	*I can remember not wanting to get high but getting high anyway...And I can remember feeling hopeless.*

**Table 2:** Chronic early recovery.

Sub-theme	Exemplar
Systemic challenges	*Trying to get housing [was the biggest challenge in my recovery process].* *I grew up in the system. I did not know nothing about work. I did not have no skills or nothing.* *Maybe one day they will have a facility that will help people of color the same way that they help people of lighter color.*
Incarceration	*[Recovery in prison is] a totally different animal.*
Individual challenges	*One thing that I’m so grateful for is to break the chain of addiction in my family.* *I was not the type of man to share my innermost thoughts and feelings. I thought that it hindered my masculinity.*
Inadequate support	*[The therapist] was judging me off why I was there versus trying to figure out who I was.*

**Table 3:** Recovery pathways.

Sub-theme	Exemplar
Mutual aid groups	*You can talk and receive and give information to people actually listening and applying it.*
Treatment pathways	*[I tell people new to recovery to] go into treatment first. Then after treatment, go to a recovery house.*
Religion/spirituality	*My recovery comes from me having faith…God is the major factor in my recovery.*
Blended pathways	*For me, it’s prayer, [Bible] reading, going to meetings, getting a sponsor, working the steps.*

**Table 4:** Facilitators of recovery vary by recovery stage.

Sub-theme	Exemplar
Initial and early recovery	
Receiving support	*My mother, she keeping me staying [in recovery]. She like, “Baby, you doing good...You handling your business. I got your back.”*
Peers with shared identity	*I do an AA group…a women’s meeting...It just be us women talking and encouraging each other.*
Providers with shared experience	*[My recovery coach and I] grew up on the same side of town. And I really connected with him.*
Cutting ties with negative influences	*Some of the people that you love, you have to cut them off...you do not want them to put you in a situation where you relapse.*
Sustained and stable recovery	
Being a caregiver	*My son, he’s 13 now, but he like, “Pops, I need you back in my life...”.That’s motivating me to stay on the right track.*
Providing support to peers	*If you see that you are helping the new person, it helps you.*
Repairing damaged relationships	*Me and my mother has mended our relationship through my sobriety.*

### 3.1 Delayed recovery initiation

Participants described spending years, decades in some cases, in active addiction before they entered recovery. A common sentiment in participant stories that may help explain delays in recovery initiation was that many participants had neither a vision for what recovery might look like, nor the belief that it was possible. This was due to an intersection of factors, reflecting systemic, community, and individual challenges, including the following sub-themes. See Table 1 for a summary of these sub-themes and exemplar quotes.

#### 3.1.1 Systemic challenges

The systemic challenges participants described related to low socioeconomic status (SES), structural racism, and environmental trauma. Many reported a causal chain of experiences that began with growing up in poverty, then engaging in activities such as selling drugs or sex work for economic survival, which often led to legal system involvement. Due to an impoverished living environment, they felt those in their community did not have a choice to opt out of this causal chain:

*I knew a lot of people that their whole family sold dope. They really did not have a chance to do nothing else but sell dope. Even though if they went to school or whatever, smart as a whip, they still sold dope because that was what their people did. That’s what their people did to make money* (participant #5).

Some commented on the structural racism that resulted in their growing up in poor, urban neighborhoods. This participant explains that as a Black man in the United States, he was at a societal disadvantage, so he felt it was essential to keep his mind sharp to survive. Alcohol and drugs threatened his mental clarity and focus:

*On the south side of every city is where you are going to find poor Black folk… [The system] is set up for failure…It’s all set up for us, man. Basically, I was born with the cards dealt against me already…So, you need to be focused, and you need to try to do the best you can. And when you have anything that’s altering your thinking, no matter if it’s marijuana or alcohol, you might not make the right decision* (participant #6).

A prevalent characteristic was the experience of environmental trauma, which stems from unstable or unsafe situations in which people live (e.g., homelessness, community violence, family incarceration), as distinguished from personal traumatic events (e.g., abuse). While describing their background and recovery stories, most (81%) reported experiencing environmental trauma at some point in their lifetime. Several articulated the causal connection between trauma, race, and substance use. For example:

*Depression, anxiety, trauma goes a lot in the black community. So that’s a lot that we deal with, and that’s a reason why people turn to drinking, or drugs, or whatever to cope. But at the end of the day, them problems still there* (participant #10).

#### 3.1.2 Omnipresence of alcohol and drugs

Participants described living in environments where cues for alcohol and/or drug use were plentiful. A prevalent environmental theme was the omnipresence of alcohol. Most reported growing up in, and currently living in, neighborhoods that were poor and majority Black. Many described the abundance of liquor stores in these neighborhoods and the challenge of facing down this persistent enticement, even when positive supports, like the church, were available:

*You got a liquor store on every corner and the church, so you got to choose one…I mean, it is quick to go to the store. Because you are going to the store anyway, and then if you a drinker your mind be like, “Ah, I need a shot because my day ain’t been good.” And you go and get a shot. Maybe it’ll calm me down or I forget about the situation, but nah* (participant #26).

Participants also discussed the prevalence of alcohol in their social environment. When asked about substance use in their social networks, almost all (92%) reported having family members or friends who used around them. This was reported as well by several veterans as common in the military, where they encountered a heavy drinking culture. Drug dealing and drug use was also prevalent in their physical and social environments. Many described the challenge this presented to initiating recovery.

When asked about their substance use over time, nearly three-quarters (73%) reported polysubstance use and/or switching substances at some point. Some articulated that polysubstance use and/or switching extended their years of active use and delayed recovery:

*I went from alcohol to marijuana…went from marijuana to cocaine. I did the cocaine for quite a few years…I really did not stop the cocaine because I went from smoking rocks to using powder…And I then started popping ecstasy pills. I then went to meth, and this is when I seeked recovery…So, I had a drug span and an addiction and substituted through drugs for many years* (participant #13).

#### 3.1.3 Individual challenges

Many individual challenges participants experienced were related to individual trauma and/or gender norms. Individual trauma is characterized by a specific upsetting event or series of events (e.g., rape/sexual assault, police violence, death of a loved one). While revealing their background and recovery stories, most (84%) reported experiencing individual trauma in their lifetime. Of note, many participants’ trauma had an intergenerational nature. That is, the effects (e.g., emotional pain) of the individual and/or environmental trauma experienced by family members (e.g., systemic oppression, child abuse) got passed down to participants. Sometimes this took the form of caretaker instability or absence, a challenge that had long-lasting effects that participants had to overcome to initiate recovery:

*I had a really rough childhood, so I just know I do not want that for my child. So, whatever I got to do to keep myself together and that’s pretty much what I’m on…[I’m] trying to break that [cycle] completely…I should not have had to grow up on my own and figure things out on my own. I should have had somebody, had my parents there* (participant #2).

Female participants reported that engaging in sex work was both a facilitator of and a product of their substance use. Several women described getting stuck in a cycle of sex work and polysubstance use, and recovery initiation was delayed for them due, in part, to this sex work-substance use cycle:

*I know women stay longer out there using…If you are out there selling your body for somebody, they want to keep you selling your body, so they going to keep giving you more alcohol and more crack. Men do not sell their bodies as much as women do, so we are out there longer. We have more people trying to keep us out there longer* (participant #8).

Women also discussed the caregiving and household responsibilities that fell on them. This resulted in many choosing to hide their substance use because they could not take time away from their families to seek treatment. As a female participant explains, this is another reason women may be less likely than men to seek help:

*I think women are scared to come out and seek help. Because they do not have that help or they want to hide the pain, do not want their significant other to know or their kids to know that they really struggling. So, you’ll have more men to go get the help than females. That’s what I believe* (participant #11).

#### 3.1.4 Lack of role models and hopelessness

Another common theme was a lack of healthy role models in their lives. As mentioned above, substance use was prevalent in almost every participant’s social network. This female participant discusses how learning to live a healthy life took her a long time because she did not have adequate role modeling:

*When you are a little girl, you grow up feeling like you have to do all these things you have seen. And now that I realize that, no, I can be a healthy mom, and this is what that looks like. I do not have to go out there and share myself out of wedlock like that with men. And no, love does not mean abuse. Learning how to be a young woman was something that took a long time because I just did not have those role models* (participant #27).

This lack of guidance also resulted in limited exposure to witnessing other people in recovery living improved lives. Some participants went a long time without being introduced to the concept of recovery. This led many to feel hopeless about ever being able to change course and find a pathway to recovery:

*For a long time, I never knew recovery existed…I had no idea in the throes of my addiction that there was a place to get some help for a long time* (participant #20).

*I can remember not wanting to get high but getting high anyway...I told God, “If you take this away from me, I’ll never do it again.” As soon as somebody knocked at the door, had an eight ball, I forgot all about the pain, and just smoked and smoked and smoked. I can remember the inability to say, “No,” not only to drugs, but to sex. For me, sex and drugs was hand in hand. They went hand in hand. And I can remember feeling hopeless* (participant #18).

### 3.2 Chronic early recovery

Once participants entered recovery, our findings reinforced a recovery stage model proposed in the SUD recovery literature—*initial, early*, *sustained*, and *stable*. However, many reported struggling with chronic relapse, which resulted in prolonged time in the *initial* and *early* stages of recovery, stunting progress toward stability:

*I was a chronic relapser. I could get a little time clean. I could get six months; I could get three months. I was constantly doing that throughout the years. And I looked up, and the years were going by and by and by* (participant #33).

The following sub-themes characterize the ways participants became stuck in early recovery. See Table 2 for a summary of these sub-themes and exemplar quotes.

#### 3.2.1 Systemic challenges

Participants reported significant socioeconomic challenges to recovery progress, such as not being able to find adequate and affordable housing, lacking access to transportation to attend recovery meetings, lacking training and job skills for employment, and feeling unsafe in one’s living environment due to neighborhood criminal activity. The prevalence of drugs and alcohol in their neighborhoods and social networks, which served as a barrier to recovery initiation, also affected their ability to achieve stable recovery.

Many described direct and indirect experiences with institutional and/or structural racism that were traumatic and affected their recovery trajectory. These environmental traumas included inequitable treatment by the criminal justice system. Many talked about the intersection of class and race that resulted in having less access to high-quality SUD treatment and recovery resources for themselves and those in their communities, as compared to those in high-SES white communities:

*I know it would never be even as far as white people getting help and black people getting help, because that’s just the way it is. But maybe one day they will have a facility that will help people of color the same way that they help people of lighter color* (participant #17).

#### 3.2.2 The role of incarceration

Many had been incarcerated and described their time in jail or prison as being part of their relapse cycle. That is, relapsing would often lead to illegal behavior (e.g., drug possession, drug dealing, larceny), which would then lead to criminal-legal contact and incarceration. Some attempted to recover from AUD in prison which presented unique challenges to the recovery process. One of the biggest was community reentry, a common point of relapse (63–65) due to the fresh, overwhelming threats to one’s recovery. One participant who had recently reentered the community after a five-year prison term described recovery while being incarcerated as “a totally different animal.” He felt recovery in prison was easier due to fewer enticements; it was a good start, but continuing one’s recovery upon reentry would require a different level of discipline and support:

*I’m grateful for the little five years [of recovery] I got right now, even though I did it in prison. But I’m here to try to gain the same foundation out here that I did in prison* (participant #5).

#### 3.2.3 Individual challenges

As with the theme of delayed recovery initiation, many discussed individual trauma as a significant barrier to overcome on their road to sustained and stable recovery. This included attempting to break the cycle of intergenerational addiction and dysfunction in their families:

*One thing that I’m so grateful for is to break the chain of addiction in my family because on both sides, everybody is addicted, whether it’s pills, heroin, alcohol, relationships, everybody, everywhere. I mean to this day. So, when you are in an environment where everybody’s doing things, you wonder if that’s what you ought to do* (participant #27).

Both men and women noted that challenges relating to gender norms impacted their early recovery. Men reported being uncomfortable sharing personal thoughts, feelings and experiences from their lives, a practice which is encouraged in most recovery settings. Women stated that because many recovery settings were male dominated (e.g., AA), they often felt vulnerable and worried that men would take advantage of them. Participants saw these challenges as being threats to their recovery:

*Communication is the most effective tool I have against active addiction coming back again in my life. I learned to do that, and it was something different. I was not the type of man to share my innermost thoughts and feelings. I thought that it hindered my masculinity* (participant #9).

*[In early recovery] I was still looking for love, acceptance. I was a people pleaser. I was in a lot of relationships. When I stopped using drugs, I did not stop having sex. So, I had a lot of sex, with a lot of different people... [In the 1980s] the old timers [in AA] took advantage of the newcomers. They knew we were desperately seeking love and attention, and they just took advantage of us [women]* (participant #18).

#### 3.2.4 Inadequate support

Those in the *initial* and *early* stages of recovery are particularly vulnerable to relapse. Thus, it is a time when instrumental, emotional, and informational support from friends, family, and providers can be particularly beneficial. However, many participants described receiving inadequate support from their social network and/or providers in these stages. This participant described lack of support in early recovery causing her to feel abandoned and alone, which then led to relapse:

*I felt not a lot of support. I think I had some abandonment issues…I knew within myself that I was relying on the cocaine to fill some void that I could not understand…I’m raising the children. I had some issues with my marriage. And I do not know, I just felt alone, very alone* (participant #36).

Another participant noted disappointment in the support offered by their therapist:

*Before I went to DRP, I was seeing another therapist. He wasn’t Black, he was white. I felt like he was judging me off why I was there versus trying to figure out who I was…He was basically trying to tell me in so many words, “Well, you are an alcoholic, you have a problem.” Instead of really trying to find where it came from* (participant #2).

### 3.3 Recovery pathways

While many participants used conventional recovery pathways (e.g., mutual aid groups, counseling), most used more than one pathway, sometimes simultaneously, and found ways to adapt common pathways to their recovery preferences. See Table 3 for a summary of the sub-themes and exemplar quotes for the recovery pathways used.

#### 3.3.1 Mutual aid groups

When asked about the recovery pathways they used, almost half (46%) reported that AA and/or Narcotics Anonymous (NA) was at least part of their recovery journey. Many reported having positive and impactful experiences with 12-step mutual support groups:

*The main thing in recovery is your sponsor and making [mutual support] meetings, and them are the main things in recovery. If you have got them, you can do it* (participant #22).

Moreover, most (70%) reported past or present membership in a non-12-step recovery group when asked about recovery pathways used. This was likely because this type of group was a central programming component at DRP, from which they were recruited. Many noted that the groups were especially important in their early recovery. This participant described their non-12-step recovery group feeling like a supportive family:

*[At the groups] you receive information, and everybody’s honest. They speak about their problems, share their feelings and show their emotions, so it’s good. It’s like a family, and it makes you feel comfortable to have somewhere to sit. You can talk and receive and give information to people actually listening and applying it* (participant #31).

#### 3.3.2 Formal treatment

Nearly half (46%) indicated using individual counseling when asked about their recovery pathways. Some reported they felt more comfortable sharing personal things about themselves in a one-on-one setting, as compared to a group. Other recovery pathways included inpatient treatment (46%), sober housing (19%), psychoactive medication (19%), recovery coaching (14%), and mindfulness practices (3%). A common pathway progression from *initial* into *early* recovery was starting with inpatient treatment, joining a mutual aid group, perhaps seeking one-on-one counseling, and moving into sober housing if necessary:

*[I tell people new to recovery to] go into treatment first. Then after treatment, go to a recovery house and get some structure in your life. Stay there a couple of years until you feel that you are able to be on your own. You got to take baby steps during this and make meetings in that process* (participant #21).

#### 3.3.3 Religion/spirituality

The most prevalent pathway was religion or spirituality. All participants indicated that religion (most identified as Christian) or spirituality played a role in their recovery. While there was variance in religious or spiritual practices (e.g., prayer, church attendance, Bible study, meditation), many reported that organized religion with a traditional understanding of God was critical to their recovery:

*My recovery comes from me having faith. If I did not have faith in myself being what God wants me to be, I would not have even thought about continuing living. I would have kept drinking. I’d probably be dead right now…God is the major factor in my recovery* (participant #4).

Others did not engage with an organized religion or share a traditional understanding of God. Instead, they leaned on personal spiritual belief systems to support their recovery.

#### 3.3.4 Multi-pathway recovery

Participants articulated a belief in multiple pathways to recovery, countering a historical notion that 12-step groups were the only pathway. When asked about their recovery pathways, nearly all participants (95%) reported using more than one of the pathways listed above; most (81%) reported using three or more of the pathways. Many said the most important thing in recovery was to find a circle of support, but there was more than one way to find it. This participant, like many, described using a blended pathway, combining prayer and Bible study with a 12-step program:

*The most helpful for me was when I finally got an understanding of the disease that I have…That’s what I identify alcoholism as…And if I do not take the medication for that disease, it’s not a matter of if I’m going to drink again. It’s a matter of when…The medication is different for everybody. For me, it’s prayer, [Bible] reading, going to meetings, sharing, getting a sponsor, working the steps. So, you got to figure out your medication, then you got to do it daily* (participant #32).

Some described struggling with established norms in certain pathways and needing to find an adaptation that better fit their recovery. One participant struggled with AA’s abstinence-based orientation but eventually found a meeting that was welcoming to their harm reduction approach. Another described struggling with the traditionally religious component of a 12-step program but was able to find a “God of my own understanding”:

*I had a very difficult time in [early] recovery because I considered myself atheist…I felt bad because everybody else was talking about God....It took me a couple of decades to find a God of my own understanding* (participant #33).

### 3.4 Facilitators of recovery vary by recovery stage

Participants described myriad facilitators of their recovery with variation according to recovery stage. Receiving support was the predominant theme in the earlier stages, while finding meaning was more predominant in the later stages. The following sub-themes describe these facilitators. See Table 4 for a summary of sub-themes and exemplar quotes.

#### 3.4.1 Initial and early recovery

The most critical facilitator in early recovery, receiving support, came in differing forms and from a variety of people. Participants received three types of support: instrumental, emotional, and informational. The support came from long-standing members of participants’ social network (e.g., family and friends), those new to their social network (e.g., peers in recovery), and/or those outside their social network (e.g., professionals):

*My mother, she keeping me staying [in recovery]. She like, “Baby, you doing good. You looking good. Everything going good for you. You handling your business. I got your back. You ain’t got to worry”* (participant #12).

*Here at DRP, they are very helpful. Showing me, teaching me. [I’m] learning the process of being in recovery and identifying some of my behaviors. They helped open my mind to see that what I was doing was insane* (participant #16).

Female and LGBTQ+ participants, in particular, reported that receiving support from peers with a shared identity was important to feel comfortable and safe in their early recovery:

*I do an AA group through Zoom…[It] is a women’s meeting...It just be us women talking and encouraging each other…It’s a sisterhood of those who use alcohol as a friend, as a crutch and no longer want to do it no more* (participant #29).

The background of providers (e.g., counselors, peer recovery coaches) also affected success in early recovery. Providers with shared experience made participants feel less judged and more at ease:

*A lot of the instructors at DRP were ex-addicts too. One was a [recovery coach]. We grew up predominantly on the same side of town in the same areas. And I really connected with him because he was a guy like me who sold drugs* (participant #13).

Many reported that it was also important to cut ties with negative influences for success in early recovery. Painfully, for some, this meant eliminating or reducing contact with loved ones, such as family members and close friends:

*Some of the people that you love, you have to cut them off because you do not want them to put you back in a situation where you relapse. That was my father. He drinks, but if he was to start drinking around me, it’s time for me to leave his house* (participant #14).

#### 3.4.2 Sustained and stable recovery

To sustain recovery, many discussed the importance of finding or re-finding meaning and purpose in their lives. They did this in a variety of ways. They pursued education and/or employment, which one participant described as the pathway to becoming a “productive member of society.” Many in stable recovery reported that they now worked with others in active addiction or initial recovery in a professional capacity (e.g., peer recovery coach, hospital staff). Some reported that providing support to others in their life, whether in a professional capacity or not, was also a facilitator of sustained recovery. This included being a caregiver, usually to children, and/or providing support to one’s peers in recovery groups:

*My son, he’s 13 now, but he like, “Pops, I need you back in my life.” Because his mother, she not really doing too good, so he need me. That’s another thing that’s motivating me to stay on the right track and keep me going stronger* (participant #12).

*My understanding of [AA] is how you help other people is you tell your story…And by you continuously carrying the message, it helps the new person. If you see that you are helping the new person, it helps you. It has a reciprocal type thing* (participant #15).

For many, repairing damaged relationships with loved ones, often parents and/or children, was an essential component of sustaining recovery:

*Now, me and my father has gained a relationship through my sobriety. Me and my mother has mended our relationship through my sobriety…When I seeked help and counsel through [my father]...we built a relationship through our common struggles. And my mother...when she seen me trying to get myself together, she was proud of me* (participant #1).

## 4 Discussion

Our study provides valuable insight and expands the understanding of recovery pathways from AUD for Black individuals within a low-income urban setting, stemming from the rich and nuanced stories shared by these participants. We identified themes related to systemic societal challenges, interpersonal supports and role modeling, and personal challenges, among others. Some barriers and facilitators to recovery initiation and recovery maintenance, such as receiving support from a social network, repairing relationships or cutting ties with negative influences, and finding or re-finding meaning and purpose in one’s life, are recurring themes in the recovery literature (28, 66–69).

However, there were nuances and components specific to Black populations that are new contributions to our understanding of recovery. Participants described experiencing interpersonal, institutional, and structural racism which affected their housing, employment, and sense of safety. Such threats, or adverse determinants of health, resulted in delayed recovery initiation and/or chronic early recovery for many participants. Yet, these participants successfully attained recovery, and some reached the later stages of stable and sustained recovery, using a range of pathways.

In recovery, participants were greatly involved with religion and/or spirituality, reflecting other literature that emphasizes the importance of religion and spirituality in Black populations (50, 52–54, 59). This was the only shared recovery pathway among all participants. However, there was variance in how people integrated religion and spirituality into their recovery. Some were part of active, community-based religious groups (e.g., church, Bible study); others engaged in private spiritual practices (e.g., prayer, mindfulness). Of note, a minority identified as atheist, yet they found a way to make the “higher power” of AA work for them by finding a “God of their own understanding,” an example of adapting a recovery pathway to one’s preference. For many, this religious/spiritual pathway was blended with a conventional pathway such as mutual support groups and individual counseling, again reflecting what we know about recovery pathways across populations (33, 70–72).

Many participants indicated that the race and background of peers in recovery and treatment providers influenced their recovery journey. They felt more comfortable attending groups with peers and one-on-one sessions with providers who shared at least some of their experience. Peer recovery coaches played an especially influential role for this sample. Shared experience and/or identity with peers and providers, an essential component of culturally sensitive care, may be an important protective factor especially for Black individuals in early recovery (73, 74). Race was not always the most crucial aspect of shared experience, as noted by participants (74). Growing up in a similar neighborhood and/or struggling with substance use and/or mental health may be more important shared experiences (75, 76).

This study provided aspects of recovery pathways that were specific to Black, low-income communities in participants’ own words. While they described being affected by societal challenges, including racism and trauma, it was not always clear to what extent effects such as housing instability or environmental traumas were due to race versus poverty. Many described growing up in poverty, experiencing challenges such as caretaker instability, environmental and individual level trauma, and/or racism in childhood and drew a causal connection between these factors and the appeal of alcohol or drug use. Economic pressures pushed participants to sell drugs or engage in sex work for survival, which further facilitated substance use and often led to criminal-legal involvement. Importantly, this can lead to injury, incarceration, illness, and social problems, all of which Black individuals experience disproportionally (5–8). Intergenerational experiences of trauma and addiction were additional challenges.

Participants discussed the omnipresence of alcohol and drugs in their neighborhoods, families, and friend groups, and the absence of healthy role models in their social networks as barriers to achieving and sustaining recovery. Furthermore, this led to a lack of exposure to those in recovery, which limited participants’ expectations of what was possible and increased their feelings of hopelessness. Some participants told stories of recovering in prison. However, incarceration limits engagement with one’s loved ones and community, and thus likely caps one’s ability to successfully move through the recovery stages, as well as presenting the significant threat of relapse upon re-entry to the community (77, 78). These findings lay out a complex web of barriers to recovery initiation and to maintaining long-term recovery in this low-income urban Black community that may not be felt so consistently by the general population.

Our findings on the relationship between gender norms and recovery pathways seem to support components of prior research, while presenting new insights. Reinforcing results from prior studies, female study participants reported experiencing delays in recovery initiation as compared to male counterparts for reasons including caregiving responsibilities at home, negative beliefs around prioritizing needs, and that seeking treatment might threaten important relationships (79–81). Additionally, Black, low-income women may be more at risk of recovery delay due to the intersection of factors they are more likely to encounter than the general population, such as sexual trauma, misogyny, racism, and lack of healthy role models (82, 83). However, once female participants entered recovery, they often struggled with relapse less than men. Female participants emphasized the importance of women-only recovery groups as an important facilitator.

In summary, our participants’ stories emphasized the burdens experienced by this Black community at personal, interpersonal, environmental and societal levels. Moreover, they directly connected these burdens with the difficulties they experienced in knowing about, achieving, and staying in recovery from alcohol problems. Even when social and professional supports were available, certain characteristics, such as shared experiences, were deemed more valuable than others. In addition to comments about struggles and past feelings of hopelessness, participants also expressed strength in their recovery, and appreciation for their faith and recovery-based supports.

### 4.1 Limitations

Our qualitative exploration of pathways to recovery from AUD in a Black community had a few limitations. First, selection bias was possible as most of our participants were clients at the same recovery organization. This limited the variety of recovery pathways represented in the study, with most people having used recovery coaches or formal treatment. For example, “natural recovery”—the process of recovering without formal treatment or mutual aid groups—is a known phenomenon discussed in the recovery literature (25, 84), but we did not speak to anyone who used this recovery pathway. Second, the average age of our sample was 50 years old; the youngest participant was 30, which may have reflected the population served at DRP, where we recruited participants. While we collected retrospective data from participant stories, our study was not able to capture data on recovery pathways for younger Black individuals. Third, because issues of race and class are intertwined for participants in this sample, we were limited in our ability to consider the unique effects of each. For example, structural racism has clearly affected our participants, yet we cannot always distinguish its effects from those of poverty. Finally, our study did not include individuals with high incomes or those who live outside of urban areas therefore our findings are not representative of a general Black population.

Despite these limitations, the study provides important foundational knowledge about recovery pathways in a Black population and indicates some essential differences from what is known more broadly about recovery pathways. Future research will similarly examine the understanding of recovery itself among these same participants to identify if the definitions of recovery writ large hold in this Black community. Additional research should examine these questions in a way that broadens the understanding of the heterogeneity of Black individuals in the U.S. who are striving for or achieving recovery from alcohol or drug use disorders, considering variations by SES, other determinants of health, and geography, as well as by sub-populations that we were unable to examine in depth, such as Black people who identify as LGBTQ+ and young adults.

## 5 Conclusion

Our study had two main strengths. First, the field of recovery is nascent, and most research done thus far has reflected the majority-white population of the U.S. Recovery pathways from AUD in Black communities are understudied and poorly comprehended, and we advanced the knowledge base. Second, instead of strictly attempting to understand recovery in Black communities as compared to, for example, white communities, our study was focused on exploring the heterogeneity within Black recovery pathways. Thus, we believe the findings from this study enable a deeper understanding of what recovery means to Black individuals and how it can be measured. The results have meaningful implications for clinical treatment and recovery supports for this population, including stressing the importance of trauma-informed care and flexible recovery support structures that involve robust peer recovery services. The findings also indicate the importance of addressing the determinants of health through community investment to advance the recovery journeys of Black individuals with AUD.

## Data Availability

The datasets presented in this article are not readily available to protect participants’ privacy; data presented here will not be shared. Please contact the corresponding author for additional information. Requests to access the datasets should be directed to avoseoneal@brandeis.edu.
