# Supplementary material for: Understanding pathways to recovery from alcohol use disorder in a Black community
Source: Front Public Health. 2025 May 1;13:1537059. doi: 10.3389/fpubh.2025.1537059 (PMC12078236; doi:10.3389/fpubh.2025.1537059)
Supplement: Supplementary file 1 [file Table_1.docx]

**Supplementary Material**

**UPWARD Interview Guide**

(Modified from Flaherty et al., 2014)

1. Tell me a little bit about yourself, for example, your background.

*Potential prompts*: Where did you grow up? What was it like growing up there? What did your parents or caregivers do for work? What were your hobbies? Who did you spend time with? What was your relationship with your parents or caregivers like? What was your relationship with your siblings and friends like? Who did you turn to for support?

1. Tell me about your relationship to alcohol and other substances.

*Potential prompts*: How were you introduced to alcohol and other substances? When did you begin to use substances? Where did alcohol fit in? How/when did you use alcohol (e.g., while under the influence of other substances)? When did you notice substance use being a problem for you? Before recovery, how did you make sense of what was happening to you?

1. Do you feel any of the following played a role in your struggles with alcohol and/or other substances…Your upbringing? The environment (community) in which you grew up? Alcohol/drug/mental health/other issues in your family? Your race/ethnicity? Your gender identity? (Sexual orientation? —**ask only if indicated from screening**) Employment issues? Housing issues? Legal issues? Mental and/or physical health issues? If so, please explain.
2. Tell me about your story of recovery.

*Potential prompts:* How long have you been in recovery for? How did you get to where you are today? Has your environment (community) changed since being in recovery? Have there been stages to your recovery? Have you continued to use alcohol and/or other substances in recovery? What was most helpful to you in early recovery? What is most helpful to your recovery now? Has professional treatment played a role in your recovery? What role has DRP played? Have self-help or support groups played a role in your recovery? What have the biggest challenges been in your recovery process?

1. How has your race/ethnicity played a role in your recovery? How about your gender? (Sexual orientation? —**ask only if indicated from screening**)
2. Based on your own experience, how would you define recovery?

*Potential prompts:* How do you know you’re in recovery as opposed to active addiction? How do you know you’re getting better?

1. What role have others played in your recovery – positive or negative?

*Potential prompts:* How have important people in your life (partners, family, friends, co-workers, etc.) responded to your recovery? What role, if any, has helping others played in your recovery?

1. How have the relationships in your life changed because of your recovery?
2. Has religion/spirituality played a role in your recovery? If so, please explain.
3. Is there anything we didn’t talk about that’s important for us to know?

Thanks so much for your time today!
